# Potential Toxic Levels of Cyanide in Almonds (*Prunus amygdalus*), Apricot Kernels (*Prunus armeniaca*), and Almond Syrup

**DOI:** 10.1155/2013/610648

**Published:** 2013-09-19

**Authors:** Nadia Chaouali, Ines Gana, Amira Dorra, Fathia Khelifi, Anouer Nouioui, Wafa Masri, Ines Belwaer, Hayet Ghorbel, Abderazzek Hedhili

**Affiliations:** ^1^Laboratoire De Toxicologie, Centre D'assistance Medicale et Urgente, Tunis, Tunisia; ^2^Unité de Recherche du Laboratoire de Toxicologie et Environnement LR12SP07, 10 rue Aboul Kacem Chabbi, 1008 Montfleury, Tunis, Tunisia

## Abstract

Under normal environmental conditions, many plants synthesize cyanogenic glycosides, which are able to release hydrogen cyanide upon hydrolysis. Each year, there are frequent livestock and occasional human victims of cyanogenic plants consumption. The present work aims to determine the hydrocyanic acid content in different samples of cyanogenic plants, selected from the Tunisian flora, and in the almond syrup. In order to evaluate their toxicity and their impact on the consumer health in the short term as well as in the long term, using the ISO 2164-1975 NT standard, relating to the determination of cyanogenic heterosides in leguminous plants.

## 1. Introduction

Many plants synthesize compounds called cyanogenic glycosides, which are able to release hydrogen cyanide upon hydrolysis [1]. This ability, known as cyanogenesis, has been recognized for centuries in plants such as apricots, peaches, almonds, and other important food plants [2]. There are at least 2650 species of plants that produce cyanoglycosides. Once the edible parts of the plants are macerated, the catabolic intracellular enzyme *β*-glucosidase can be released and can come into contact with the cyanogenic glycosides. This enzyme hydrolyzes the cyanogenic glycosides to produce hydrogen cyanide, glucose, ketones, or benzaldehyde [3]. Large numbers of people are daily exposed to low concentrations of cyanogenic compounds in many aliments, this exposition may imply a risk to human health.

Each year, there are frequent livestock and occasional human victims of many and widespread cyanogenic plants consumption. Most cases of cyanide poisoning are caused by the consumption of the plants which are members of the Rosaceae, Euphorbiaceae, Fabaceae, or Gramineae family [4]. Released cyanide inhibits cellular respiration of all aerobic organisms by blocking mitochondrial electron transport and preventing oxygen uptake. High exposure to this potent poison in humans may cause nausea, vomiting, diarrhea, dizziness, weakness, mental confusion, and convulsions followed by terminal coma and literally death [5].

In many Tunisian regions, milled apricot kernels are widely used as a flavoring agent in pastries and cakes, while bitter almond is used to prepare traditional orgeat syrup (almond syrup) which is very popular and widely consumed in Tunisia.

In this study, we aim to determine hydrocyanic acid content in different samples of cyanogenic plants. In order to evaluate their cyanogenic potential and their toxicity, according to ISO 2164-1975 NT standard, relating to the determination of cyanogenic heterosides in leguminous plants [6].

## 2. Materials and Methods

### 2.1. Sample Collection

#### 2.1.1. Plant Material

All samples were arbitrarily chosen among the Tunisian flora. Three different varieties of sweet almond were obtained from local nuts and dry fruit shops.

The two samples of bitter almond were obtained from two different markets in “Sfax” which is known to be the main city of bitter almond cultivation in Tunisia, and the third sample was obtained from bitter almond trees grown in the north of the country.

The samples of apricot kernels were obtained from five different areas from Tunisia, namely, “Monastir,” “Sfax,” “Sbiba,” “Morneg,” and “Tastour”.

#### 2.1.2. Almond Syrup

Five different almond syrup brands were collected from the major supermarkets and stores located in Tunisia.

#### 2.1.3. Equipment

We needed for this study a steam distillation apparatus composed by two round-bottomed flasks connected at a condenser tube, a mechanical seeds grinder, a precise electric balance, and an incubator regulated at the temperature of 38 ± 2°C.

#### 2.1.4. Reagents

All reagents were instantaneously prepared within the laboratory of toxicology. 

Solution of sodium acetate (20 g/L) adjusted to pH = 5 with acetic acid, nitric acid solution *d*_20°_ = 1.38 g/mL. Silver nitrate 0.02 N, ammonium thiocyanate 0.02 N. The colored indicator was prepared by mixing one part by a volume of nitric acid and one part by a volume of a saturated solution of iron sulfate and ammonium.

### 2.2. Method

#### 2.2.1. Measurement of Hydrogen Cyanide in Plant Material

To determine quantitatively cyanide levels in selected samples, we used an argentometric method, according to ISO 2164-1975 standard, relating to the dosage of cyanogenic glycosides in leguminous plants.

The procedure for the determination of hydrocyanic acid in plant material, consisted in an acid hydrolysis of the cyanogenic glycosides, the hydrocyanic acid released from this hydrolysis was recovered in the silver nitrate solution after a steam distillation.

Hydrocyanic acid levels were determined by titration of the excess of silver nitrate, by using a solution of ammonium thiocyanate, in an acidic medium, in the presence of the color indicator. 

The apparition of brown precipitate of ferric thiocyanate, indicated the equivalence point, when the silver nitrate was totally used up.

#### 2.2.2. Operating Method

Apricot kernels and almond samples were sun-dried, then ground finely with the mechanical grinder, previously cleaned up with distilled water and a diluted nitric acid solution.

Twenty g of the milled sample and almond syrup was weighed accurately then placed in a 1000 mL round-bottomed flask with 50 mL of distilled water and 10 mL of sodium acetate 0.02 N.

The maceration was made by putting the flask, tightly closed, in the incubator at a temperature of 38° ± 2° for 12 hours. These incubation conditions ensure the complete conversion of the cyanogenic glycosides to hydrocyanic acid.

After incubation, the round-bottomed flask was cooled in an ice bath and attached to the steam distillation apparatus.

The first round-bottomed flask must be half filled with distilled water and attached to the apparatus, on a heated plate.

The second one, which contained the macerate, was attached to the condenser tube.

Water contained in the first round-bottomed flask was heated to a boil; the steam produced was conducted into glass tubing to the second round-bottomed flask, in order to carry the hydrocyanic acid's vapors and to condense them into a liquid.

One hundred milliliters of the distillate were trapped within a mixture of 50 mL of silver nitrate and 1 mL of nitric acid 0.02 N, then immediately transferred into a 500 mL graduated flask and dilated with distilled water.

This solution was filtered and 250 mL of the filtrate was collected into a dry flask with 2 mL of a color indicator. The excess silver nitrate was titrated with a solution of ammonium thiocyanate 0.02 N until the brown precipitate appears.

All samples were treated identically. A blank test was carried out under the same conditions.

The hydrocyanic acid levels were expressed in mg/kg of dry matter using the following formula:

(1) $$\begin{array}{c} \text{HCN}\left(\frac{\text{mg}}{\text{kg}}\right)=0.54\left(V2-V1\right)*\frac{500}{250}*\frac{1000}{M}, \end{array}$$

*V*1 is the volume of ammonium thiocyanate required to neutralize the excess of silver nitrate in the sample test, *V*2 is the volume of ammonium thiocyanate required to neutralize the excess of silver nitrate in the blank test, *M* is the weight (gram) of the test sample.

## 3. Results and Discussion

### 3.1. Results

Hydrocyanic acid levels found in the apricot kernels, sweet, and bitter almond are shown in Table 1.

Hydrocyanic acid levels in almond syrup are illustrated in Table 2.

### 3.2. Discussion

#### 3.2.1. Cyanide Toxicity

**Figure 1:**
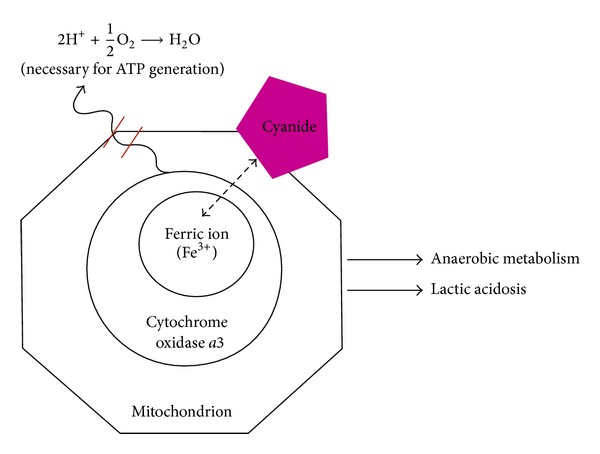
Effect of cyanide on cellular respiration: Cyanide reversibly binds to the ferric ion in cytochrome oxidase *a*3 within the mitochondria, effectively halting cellular respiration by blocking the reduction of oxygen to water. ATP: adenosine triphosphate.

Cyanide causes intracellular hypoxia by reversibly binding to mitochondrial cytochrome oxidase *a*3 within the mitochondria. Cytochrome oxidase *a*3 is necessary for the reduction of oxygen to water in the fourth complex of oxidative phosphorylation. Binding of cyanide to the ferric ion in cytochrome oxidase *a*3 inhibits the terminal enzyme in the respiratory chain and halts electron transport and oxidative phosphorylation (Figure 1) [7].

This downward cascade is fatal if not reversed. In fact, oxidative phosphorylation is essential to the synthesis of adenosine triphosphate (ATP) and the continuation of cellular respiration [8]. The toxicity of cyanide is largely attributed to the cessation of aerobic cell metabolism, which causes central nervous system and cardiovascular dysfunctions, by cellular hypoxia [9].

#### 3.2.2. Cyanide Levels in Sweet and Bitter Almonds

HCN content in the different samples analyzed varies considerably from less than 20 to more than 1000 mg/kg of dry matter. According to ISO 2164-1975 NT standard, relating to the determination of cyanogenic heterosides in leguminous plants, a sample is regarded as free from hydrogen cyanide if it contains a lower rate to 10 mg per kg; consequently, knowing that concentrations found in our samples are higher than 10 mg/kg, we consider that all treated samples are cyanogenic.

HCN levels in bitter almond (1062 ± 148.70 mg/kg) are approximately 40 times higher than levels found in sweet almond (25.20 ± 8.24 mg/kg). 

This could be explained by the fact that the amount of amygdalin contained in the bitter almond largely exceeds the amount contained in the sweet one [10]. After enzymatic hydrolysis, the amygdalin which is the most important cyanogenic glycoside in *Prunus's* species releases a high level of hydrocyanic acid, and a benzaldehyde which is responsible for the bitterness [10].

Knowing that the acute lethal dose of cyanide for mammals is as low as 0.5 mg CN/kg of the body weight, the acute oral lethal dose of HCN for humans is reported to be 0.5–3.5 mg/kg of body weight and the consumption of 50 bitter almonds is deadly for adults. However, for young children, 5–10 almonds are fatal [11].

#### 3.2.3. Cyanide Levels in Apricots Kernels

HCN levels noted in the five samples of apricot kernels vary considerably across regions of the Tunisian country. The lowest rates (583.2 mg/kg and 540 mg/kg) were noted, respectively, in samples from “Sfax” and “Tastour”. It should be noted that there are no significant differences between these two regions of North West and South East. In addition, the levels are intermediate in central Tunisia (Sbiba) with 804.60 mg/kg, while the highest levels (1134 and 1193.40 mg/kg) are noted, respectively, in samples from the Sahel (Monastir) and north of the country (Morneg).

According to The Committee on Toxicity of chemicals in food, consumer products, and the environment in the UK, concentrations of cyanide in apricot kernels can reach 2000 mg/kg of dry matter [12]. 

#### 3.2.4. Interregional Variability of Cyanide in Different Samples

The inter-regional variability of HCN content in different treated samples is mainly due to climatic conditions and rainfall. Actually, the dry climate and intense sunlight promote cyanogenesis.

In addition, agricultural areas differ by the nature of their soils and the processes of their fertilization by chemical fertilizers. Indeed, nitrogen fertilizers increase the absorption of nitrates by plants and entails blocking of nitrogen metabolism and accumulation of HCN [13]. The Age of the plant at harvest time could also explain this cyanide level variation in samples obtained from different geographic areas. In fact, it is reported that HCN gradually increases during the plant growth to reach a maximum at maturity, about 20 times higher than in plantlet [14].

A few studies on cyanogenic foods are summed up in Table 3.

According to these results, we note that, hydrocyanic acid levels in our samples of apricot kernels (851.04 ± 303.28 mg/kg), obtained by the argentometric method, are slightly lower than those found in a study conducted at the Algerian University “El Tarf” and whose object was to determine the nutritional value of bitter apricot kernels and their hydrocyanic acid levels (1175 ± 63.63 mg/kg). However, they are almost equal with a national Australian study results (799 ± 19.80 mg/kg).

Furthermore, knowing that the lethal dose is reported to be 0.5–3.5 mg/kg bw, severe toxicity would be inevitable due to the consumption of approximately 30 apricot kernels for adults and fewer for children.

According to “Committee on Toxicity” (COT), apricot kernels contain almost 1450 mg/kg of cyanide, approximately 0.5 mg/kernel. Consumers are advised to eat only five kernels in one hour and no more than 10 per day [11].

Moreover, the Canadian Health Ministry has prevented the use of bitter apricot kernels for flavoring foods or for medicinal purposes, and currently recommends that the consumption of bitter apricot kernels should not exceed three kernels a day, because of their toxicity especially for young children [17].

#### 3.2.5. Cyanide Contents in Flax Seed (*Linum usitatissimum*)

HCN levels in our samples of bitter almonds (913–1210 mg/kg) and apricot kernel (547–1154 mg/kg) are two times higher than the levels obtained from samples of flax seeds in the Australian study (360–390 mg/kg). Indeed, flax (*L. usitatissimum*), a very interesting food because of its high content of linolenic acid and dietary fiber, has the least toxicity among all cyanogenic foods. Actually, cooking flax based-food at 230°C for 15–18 minutes or boiling seeds could eliminate 90–100% of hydrocyanic acid [18, 19].

#### 3.2.6. Cyanide Levels in Cassava (*Manioc esculenta Crantz*)

The range of total cyanide contents of different varieties of cassava is 1–1550 mg HCN/Kg of fresh materiel [5]. According to the FDA, HCN content in cassava can reach up to 1500 mg/kg in bitter varieties poorly detoxified, which may explain the reported negative effects of daily consumption of cassava, such as diabetes, congenital malformations, and goiter neurological disorders such as Konzo, an epidemic paralytic disease, first described by G. Trolli in 1938, who discovered it amongst the Kwango of the Belgian Congo (now the Democratic Republic of the Congo). The outbreaks are associated with several weeks of almost exclusive consumption of insufficiently processed “bitter” (cyanide-rich) cassava. In northern Mozambique, the disease is known as mantakassa and it is induced by the daily consumption of gari (a popular food made from cassava) as a food staple, Konzo is a neurological disease which causes irreversible neuromotor damage and acute onset of paraparesis which affects mainly children [20, 21].

 Given the seriousness of this pathology, The World Health Organization has established a safety threshold of 10 mg/kg of total cyanide in cassava flour, to protect consumers against adverse effects of chronic cassava intakes [5].

 In Australia and the USA, cassava tubers were used to make chips and cookies.

#### 3.2.7. Cyanide Levels in Almond Syrup

Almond syrup's analysis shows that the five brands are substantially free of hydrocyanic acid or about 1–3 mg/kg. The very low concentrations found are most probably due to the fact that the three first brands of almond syrups are prepared with a synthetic aroma of bitter almond, that is why they contain only 1 ± 0.25 mg/kg of HCN. The two other ones are prepared with a natural aroma of bitter almond but they contain no more than 3 ± 0.5 mg/kg of HCN, probably because the amounts of bitter almond are not high enough to release significant levels of HCN.

In addition, it has been admitted that the release of cyanide only occurs after hydrolysis upon contact with water [22], in such case, cyanide was probably released during the production process of the almond syrup.

The Committee of Experts on Flavourings of the Council of Europe and Australia, New Zealand Food Standards code, have fixed regulatory limits, that define the maximum permitted levels of HCN in fruit seeds and pits-based beverages as shown in Table 4.

In light of the above results, we concluded that HCN the contents in almond syrups commercialized in Tunisia comply with the standards, So, on these drinks do not imply any dangerous effects on the human health from this point of view.

## 4. Conclusion

This study revealed a wide range of cyanide concentrations in commonly available bitter almonds and apricot kernels, in contrast to almond syrup, which is exempt of hydrocyanic acid and remains a product without any risk to human health. However, a number of recommendations should be considered to avoid the toxicity of cyanogenic foods. Emphasis should be placed on the food education, to raise awareness about the potential health risk caused by cyanogenic plants for humans, especially for children. However, the genetic selection of cyanogen-free genotypes seems to be a radical solution for this kind of intoxication. 

## Supplementary Material

Supplementary material needed for this study is 250 and 500 graduated measuring cylinders, glass filtering funnel, a filter paper and 250 ml Erlenmeyer flask.

## Figures and Tables

**Table 1:** HCN levels in apricot kernels, sweet, and bitter almond.

			Cyanide content (mg/kg)	Average levels (mg/kg)	Standard error (mg/kg)
Sweet almond		Variety1	27		
	Varieties	Variety2	32.40	25.20	8.24
		Variety3	16.20		

Bitter almond		Sfax_1_	1053		
	Origin	Sfax_2_	1215	1062	148.70
		North	918		

Apricot kernels		Tastour	540		
		Sfax	583.20		
	Origin	Sbiba	804.60	851.04	303.28
		Monastir	1134		
		Morneg	1193.40		

**Table 2:** HCN levels in almond syrups.

Almond syrup brands	Cyanide levels (mg/kg)	Standard error (mg/kg)
1	1	0.5
2	1	0.25
3	1	0.25
4	3	0.25
5	3	0.25

**Table 3:** HCN levels in some cyanogenic plants studied in Algeria, Australia, and Cameroon.

	Species	HCN levels (mg/kg)	Ref
Algerian samples	Apricot kernels (*P. armeniaca*)	1130–1220	[15]

Australian samples	Apricot kernels (*P. armeniaca*)	785–813	[4]
	Peach pits (*P. persica*)	710–720	
	Apple pits (*Malus spp*)	690–790	
	Flax seeds *L. usitatissimum *	360–390	

Cameroonian samples	Manioc (*M. esculenta*)	91–1515	[16]

**Table 4:** Maximun permitted HCN levels in drinks according to the Australian New Zealand Food Standards code and The Committee of Experts on Flavourings of the Council of Europe.

Sources	Maximum permitted HCN levels in drinks
Australia, New Zealand Food Standards code [23]	5 mg/kg
The Committee of Experts on Flavourings of the Council of Europe [24]	1 mg/kg
