# Supplementary material for: Potential Toxic Levels of Cyanide in Almonds (Prunus amygdalus), Apricot Kernels (Prunus armeniaca), and Almond Syrup
Source: ISRN Toxicol. 2013 Sep 19;2013:610648. doi: 10.1155/2013/610648 (PMC3793392; doi:10.1155/2013/610648)
Supplement: Supplementary file 1 — Supplementary material needed for this study is 250 and 500 graduated measuring cylinders, glass filtering funnel, a filter paper and 250 ml Erlenmeyer flask. [file 610648.f1.pdf]

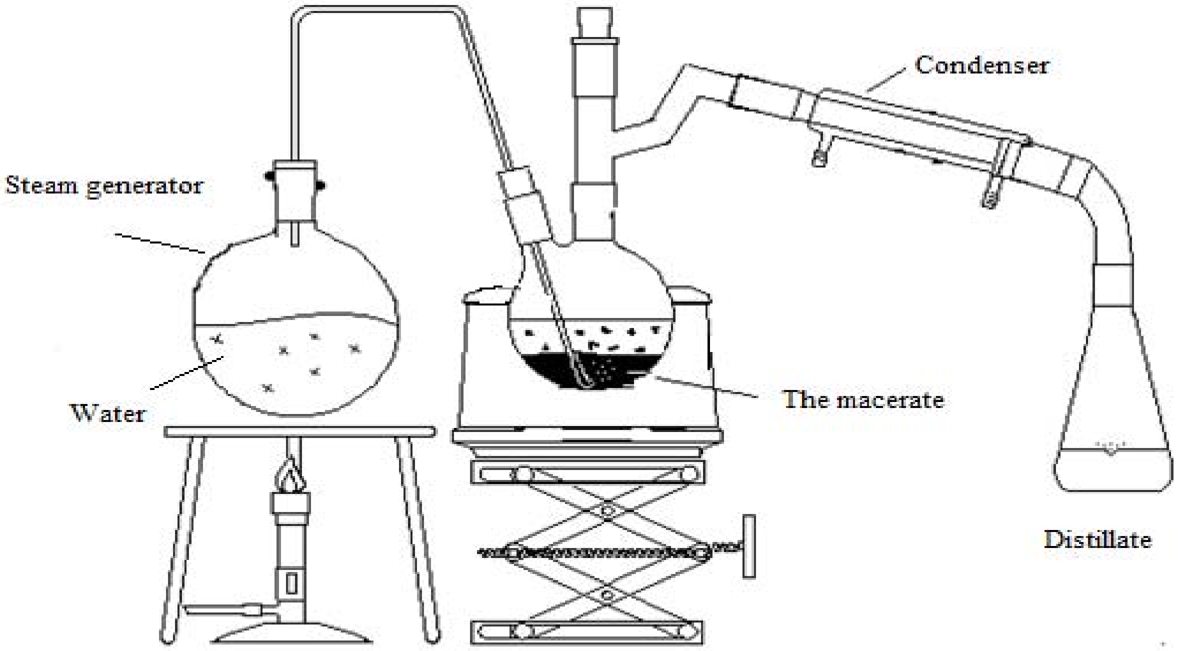

Figure1: Steam distillation apparatus; Vapors are formed by heating the water in the first round-bottomed flask, Steam produced are drawn into the glass tube, then leads the hydrocyanic acid released by maceration, in the second round-bottomed flask, vapors of HCN are condensed and trapped in silver nitrate.

and trapped in silver nitrate.
